# Multi-omic analysis of glycolytic signatures: exploring the predictive significance of heterogeneity and stemness in immunotherapy response and outcomes in hepatocellular carcinoma

**DOI:** 10.3389/fmolb.2023.1210111

**Published:** 2023-06-07

**Authors:** Shiyu Zhang, Yangting Pei, Feng Zhu

**Affiliations:** ^1^ Department of Emergency, Jincheng People’s Hospital, Affiliated Jincheng Hospital of Changzhi Medical College, Jincheng, China; ^2^ Department of Medical Record, Jincheng People’s Hospital, Affiliated Jincheng Hospital of Changzhi Medical College, Jincheng, China; ^3^ Department of General Surgery, Jincheng People’s Hospital, Affiliated Jincheng Hospital of Changzhi Medical College, Jincheng, China

**Keywords:** HCC, heterogeneity, stemness, glycolysis, immunotherapy

## Abstract

**Background:** Hepatocellular carcinoma (HCC) is a global health challenge with complex pathophysiology, characterized by high mortality rates and poor early detection due to significant tumor heterogeneity. Stemness significantly contributes to the heterogeneity of HCC tumors, and glycolysis is crucial for maintaining stemness. However, the predictive significance of glycolysis-related metabolic genes (GMGs) in HCC remains unknown. Therefore, this study aimed to identify critical GMGs and establish a reliable model for HCC prognosis.

**Methods:** GMGs associated with prognosis were identified by evaluating genes with notable expression changes between HCC and normal tissues retrieved from the MsigDB database. Prognostic gene characteristics were established using univariate and multivariate Cox regression studies for prognosis prediction and risk stratification. The “CIBERSORT” and “pRRophetic” R packages were respectively used to evaluate the immunological environment and predict treatment response in HCC subtypes. The HCC stemness score was obtained using the OCLR technique. The precision of drug sensitivity prediction was evaluated using CCK-8 experiments performed on HCC cells. The miagration and invasion ability of HCC cell lines with different riskscores were assessed using Transwell and wound healing assays.

**Results:** The risk model based on 10 gene characteristics showed high prediction accuracy as indicated by the receiver operating characteristic (ROC) curves. Moreover, the two GMG-related subgroups showed considerable variation in the risk of HCC with respect to tumor stemness, immune landscape, and prognostic stratification. The *in vitro* validation of the model’s ability to predict medication response further demonstrated its reliability.

**Conclusion:** Our study highlights the importance of stemness variability and inter-individual variation in determining the HCC risk landscape. The risk model we developed provides HCC patients with a novel method for precision medicine that enables clinical doctors to customize treatment plans based on unique patient characteristics. Our findings have significant implications for tailored immunotherapy and chemotherapy methods, and may pave the way for more personalized and effective treatment strategies for HCC.

## 1 Introduction

HCC is the most common type of liver cancer, and is responsible for a significant proportion of cancer-related deaths worldwide (Yang et al., 2019). Established risk factors for HCC include liver cirrhosis and metabolic syndrome, which have a negative impact on the prognosis and reduce overall survival rates (Llovet et al., 2016; Guo et al., 2018; Leslie et al., 2022; Huang et al., 2023). While recent advances in conventional treatments such as chemotherapy, surgery, and radiation therapy have shown promise, HCC recurrence and metastasis remain significant challenges (Sun et al., 2021). Current prognostic models for liver cancer, which rely on clinical indicators like grade and TNM stage, may have limited accuracy (Icard et al., 2021; Zhai et al., 2022; Conche et al., 2023; Wang et al., 2023). Therefore, there is a need for new biomarkers that can accurately predict survival and help identify specific therapy targets for HCC. Molecularly targeted treatments represent a promising avenue for the future treatment of hepatocellular carcinoma.

Malignant neoplasms are distinguished by their unquenchable demand for energy to fuel their growth. Consequently, cancer cells utilize a complex network of interrelated metabolic pathways (Li and Zhang, 2016; Soltani et al., 2021; Chelakkot et al., 2023). With their impressive metabolic flexibility, cancer cells can rewire crucial metabolic pathways like glycolysis to satisfy their heightened energy requirements (Vaupel et al., 2019). One instance of such metabolic reprogramming is the Warburg effect, which Warburg originally proposed in 1956. This phenomenon involves heightened glucose uptake, lactate accumulation, and increased ATP synthesis in cancer cells (Warburg, 1956; Vaupel and Multhoff, 2021). Aerobic glycolysis, which is a key characteristic of the Warburg effect, not only facilitates the proliferation of cancer cells, but also promotes invasion and metastasis by creating an acidic microenvironment (Huang et al., 2021). Several types of cancer, including breast, pancreatic, and gastric cancers, exhibit this phenomenon (Hu et al., 2019; Wu et al., 2020; Yang et al., 2021). While the role of aerobic glycolysis in the initiation, progression, and pharmacological management of HCC has been widely studied (Feng et al., 2020a; Feng et al., 2020b; Zhang et al., 2020; Liu et al., 2021), the prognostic relevance of genes involved in glycolysis remains poorly understood.

In this investigation, we analyzed clinical and sequencing data from the TCGA-LIHC database to explore the potential correlations between gene expression markers (GMGs) and the survival outcomes of HCC patients, as well as the genetic changes associated with these outcomes. Through LASSO analysis, we identified eleven GMGs that demonstrated robust associations with HCC. Subsequently, utilizing the cumulative weights of these GMGs, we performed patient stratification to classify individuals into high- and low-risk groups, revealing contrasting immunological landscapes and stemness features between the two groups. Notably, our *in vitro* assays employing GMGs as predictors of chemotherapy sensitivity yielded a high degree of predictive accuracy. Taken together, our results present a novel predictive framework for HCC that may facilitate the creation of customized treatment plans tailored to individual patients’ unique risk profiles.

## 2 Materials and methods

### 2.1 Acquisition of data from the TCGA portal

The Cancer Genome Atlas (TCGA) is a comprehensive database of genomic cancer information for 33 cancer types, integrating gene expression patterns and clinical data (Wang et al., 2016; Chi et al., 2023a). We accessed the TCGA database (https://portal.gdc.cancer.gov/) to obtain HCC data relevant to our study. The transcriptomic data of 374 HCC samples and 50 normal samples were incorporated in The Cancer Genome Atlas Liver Hepatocellular Carcinoma (TCGA-LIHC) dataset. Following the selection criteria of complete survival information and exclusion of duplicate HCC patient IDs, a total of 370 samples with both survival information and corresponding transcriptomic data were retained for subsequent analyses.

### 2.2 Retrieval of GMGs

The Molecular Signatures Database (MsigDB) provides access to 200 glycolysis-related genes (Liberzon et al., 2015; Zhang et al., 2023).

### 2.3 Identification of a prognostic GMGs signature

Our univariate Cox regression analysis identified 45 genes significantly associated with HCC patient survival. The least absolute shrinkage and selection operator (LASSO) method is a type of shrinkage estimation (Wang et al., 2022a). It constructs a penalty function to obtain a more refined model, which compresses some coefficients and sets certain coefficients to zero. Therefore, it retains the advantages of subset shrinkage and provides a biased estimation for handling data with multicollinearity, simultaneously achieving variable selection during parameter estimation. It effectively addresses the issue of multicollinearity in regression analysis, with the model reaching optimal performance when the lambda value is minimized. In our analysis, the LASSO regression algorithm was employed for feature selection, utilizing 10-fold cross-validation and the R package glmnet for the analysis. Furthermore, a larger AUC value and a smaller Log-rank *p*-value indicate better predictive performance. For model selection, we extracted the number of genes whose survivalROC yielded an AUC result above 0.7. If no genes reached this threshold, we selected the gene model with the maximum AUC value. Through LASSO regression analysis, 10 core GMGs formed the basis of a risk signature. We determined patient risk scores based on their unique gene expression profiles (Chi et al., 2022a; Wang et al., 2022a). Riskscore = G6PD*0.0777 + CENPA*0.0751 + KIF20A*0.0242 + HMMR*0.0474 + STC2*0.1032 + SAP30*0.0179 + RARS1*0.1817 + B3GAT3*0.163 + TALDO1*0.0135 + EFNA3*0.0412.

### 2.4 Evaluation of infiltrating immune cells

We utilized the ssGSEA and CIBERSORT R packages (Chi et al., 2022a; Zhao et al., 2023a) to evaluate immune cell infiltration. Using the CIBERSORT algorithm, we generated immune cell type scores for each tissue sample and assigned a score to each sample based on inferred immune cell type scores.

### 2.5 Prediction and validation of chemotherapy response

We utilized the “pRRophetic” R package to calculate IC50 values of drugs to predict and confirm treatment efficacy in HCC cells (Chi et al., 2022b; Chi et al., 2023b). Sensitivity of the drugs to HCC cells was then determined using the CCK-8 test (Liu et al., 2023).

### 2.6 Analysis of functional enrichment

Functional enrichment analysis was conducted using the GSVA method and the “c2. cp.kegg.v7.4. symbols.gmt” database (Hanzelmann et al., 2013; Zhao et al., 2023b).

### 2.7 Statistical analysis

Statistical analysis was performed using the R 4.1.3 program. The significance level was set at *p*-values <0.05 and False Discovery Rates (FDR) (q) < 0.05. The results of the Student’s t-test were presented as mean and standard deviation (SD) for the two groups. We used *p* < 0.05*, *p* < 0.01**, and *p* < 0.001*** as the levels of statistical significance.

## 3 Results

### 3.1 Gene signature construction

**FIGURE 1:**
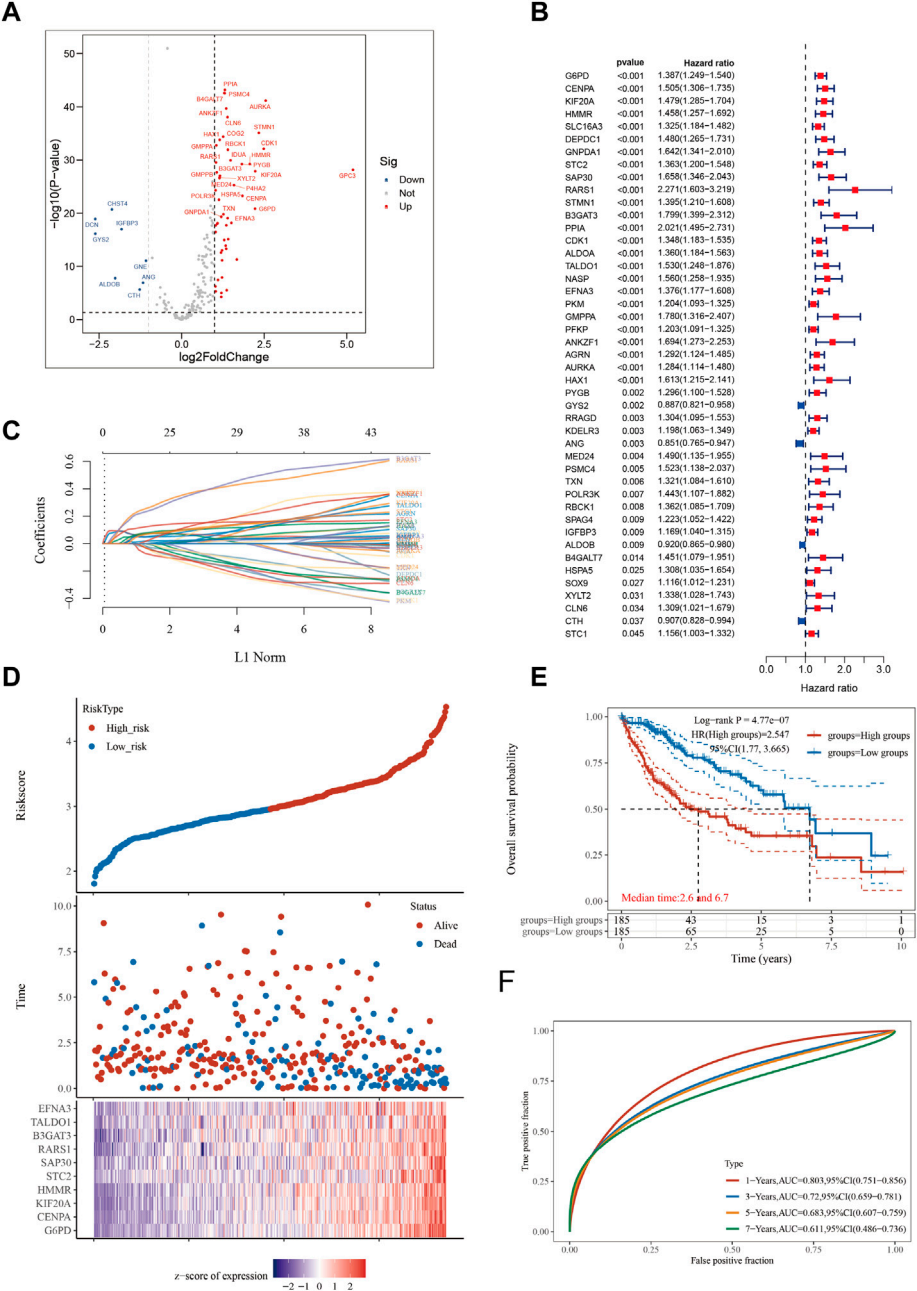
Constructing a prognostic model based on glycolysis-related genes in HCC. **(A)** Differential gene screening was conducted to identify GMGs associated with hepatocellular carcinoma (HCC). **(B)** 45 genes of prognostic significance, which we refer to as GMGs, were identified from the differential gene screening analysis. These GMGs demonstrated an association with survival in HCC patients. **(C)** Utilizing the Lasso method, a prognostic model was constructed based on the identified GMGs. **(D)** The risk scores, survival status, and expression levels of the top 10-GMGs were plotted to visualize the distribution of prognostic risk. **(E)** Kaplan-Meier (KM) analysis was performed to further investigate the prognostic significance of the 10-GMGs in different HCC subtypes. **(F)** The predictive efficiency of the prognostic model was evaluated using ROC analysis.

WWe aimed to construct a gene signature related to glycolysis metabolism by utilizing the Glycolysis_Hallmark gene collection, consisting of 200 genes, obtained from the MsigDB website. In this study, we utilized 55,316 gene expression profiles from the TCGA database, which included 370 tumor samples and 50 samples of nearby normal tissue, as well as information on HCC. Using the “limma” R package and applying a logFC filter of 1 and adj.P. Val. Filter of 0.05, we identified 59 GMGs that were differentially expressed in HCC tumor and nearby normal samples (Figure 1A). Additionally, using the “survival” and “survminer” R packages, we identified 45 GMGs that were significantly associated with patient survival at *p* < 0.05 and km score <0.05 (Figure 1B), enabling us to investigate the potential impact of GMGs on HCC patient survival. Notably, with the exception of four GMGs, all others acted as unfavorable prognostic indicators. A lasso analysis was performed using the 45 GMGs to develop an HCC predictive model (Figure 1C), which was validated by a time-dependent ROC curve showing high accuracy at one, three, five, and 7 years (AUC = 0.803, 0.72, 0.683, and 0.611, respectively) (Figure 1F). Subsequently, based on the median Riskscore, we stratified the 370 HCC patients into two distinct subgroups, high-risk and low-risk, and found that the high-risk group had significantly shorter overall survival time compared to the low-risk group (Figure 1E), with median survival times of 2.6 and 6.7 years, respectively. Finally, we generated a heatmap to illustrate the expression patterns of the top ten GMGs across different Riskscore groups (Figure 1D), providing additional support for the prognostic significance of our GMG signature in the evaluation of HCC.

### 3.2 Analysis of HCC subtypes

**FIGURE 2:**
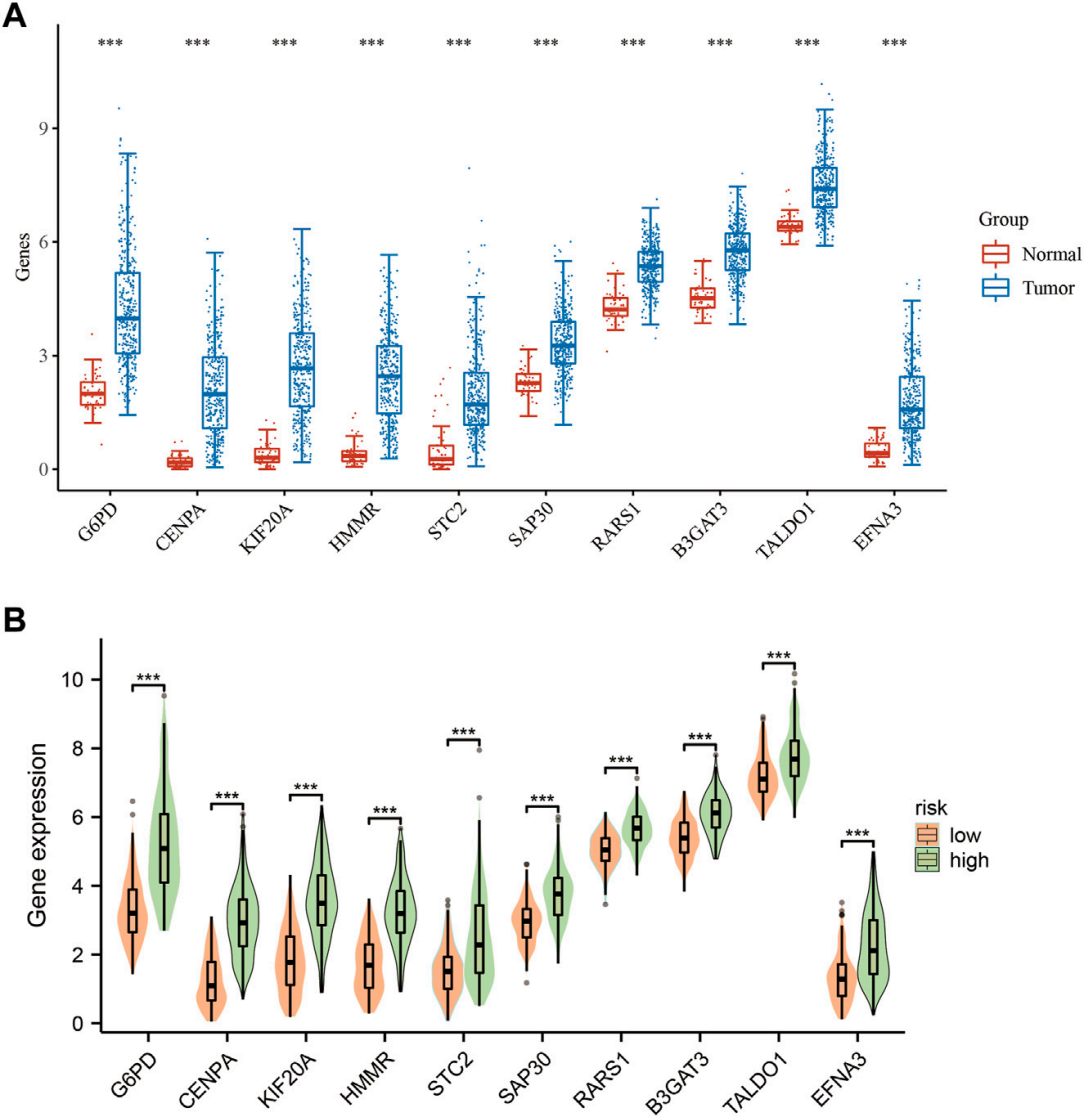
Expression levels of 10-GMGs. **(A)** Expression levels of 10-GMGs in HCC tumor tissues and adjacent tissues. **(B)** Expression levels of 10-GMGs in HCC risk subgroups. (*p < 0.05, **p < 0.01, ***p < 0.001).

To investigate the mRNA expression patterns of the 10 GMGs, we performed a comparative analysis of their expression levels in normal and tumor groups (Figure 2A). Notably, we observed that the expression of these genes was significantly elevated in tumor tissues when compared to adjacent non-tumor tissues (*p* < 0.001), with TALDO1 exhibiting the highest level of expression. The mRNA expression patterns of the 10 GMGs in high-risk and low-risk categories (Figure 2B) were consistent with those depicted in Figure 1A. To evaluate the prognostic value of each GMG, we generated Kaplan-Meier curves (Supplementary Figure S1) and found that all 10 GMGs were significantly associated with unfavorable clinical outcomes (*p* < 0.05). Further studies are required to elucidate the molecular mechanisms underlying the dysregulated expression of these genes in HCC and explore the potential for developing novel therapeutic interventions.

### 3.3 Enrichment analysis

**FIGURE 3:**
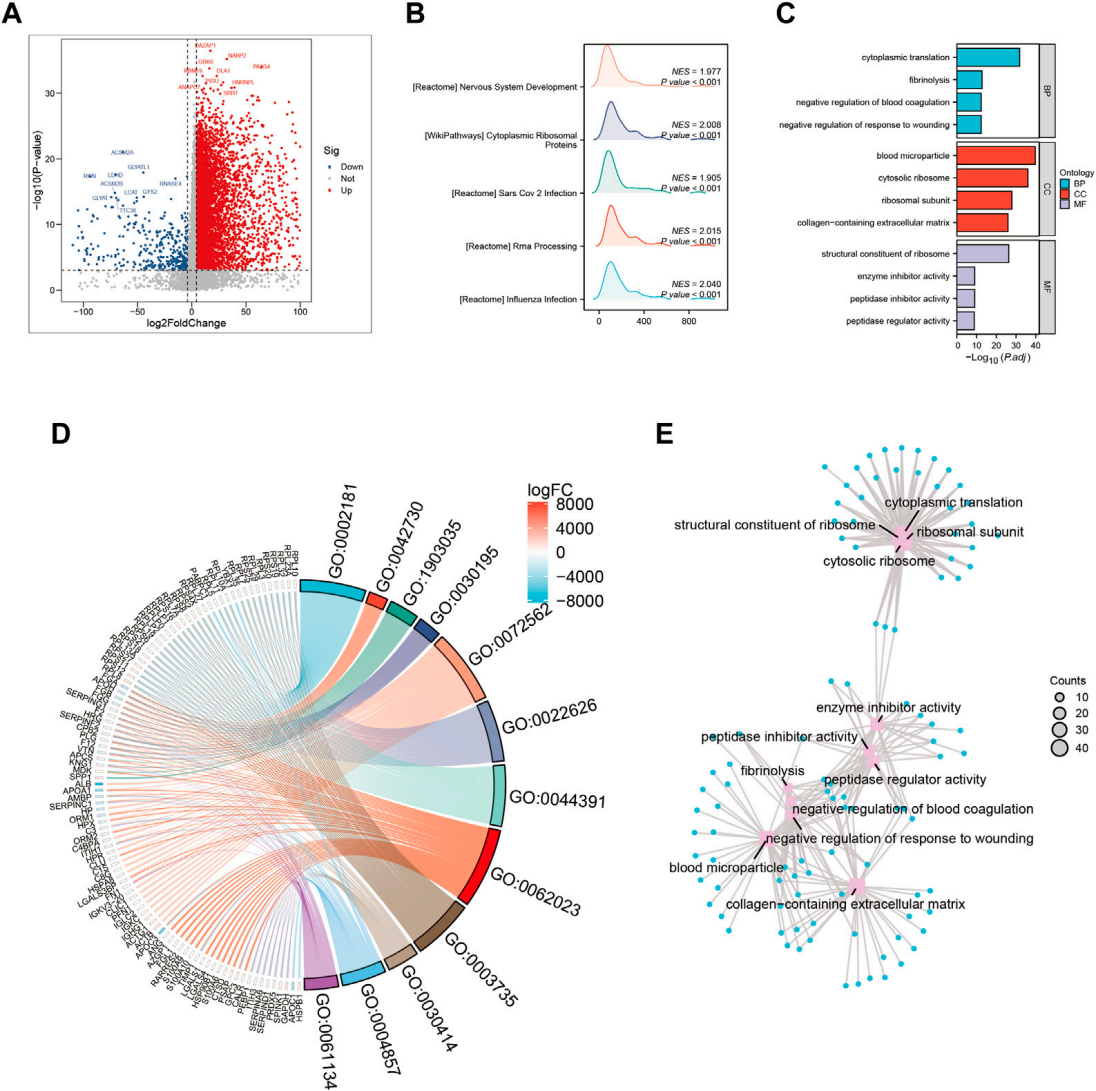
Gene ontology (GO) and Kyoto Encyclopedia of Genes and Genomes (KEGG) analysis **(A)** Volcano map screening for differential genes. **(B)** Mountain map showing the enriched KEGG pathway. **(C–E)** GO enrichment analysis.

Dysregulation of various signaling pathways is known to contribute to altered tumor microenvironments and tumorigenesis. In this study, we compared gene expression levels between high-risk and low-risk groups (Figure 3A) to identify differentially expressed genes. Our analysis revealed a significant enrichment of pathways related to cytoplasmic processes in high-risk HCC patients (Figure 3B). Furthermore, gene ontology (GO) enrichment analysis in the high-risk group demonstrated a marked activation of the biological process of cytoplasmic translation (Figure 3C). This pathway was significantly overrepresented in the differentially expressed genes between high-risk and low-risk groups (Figures 3D,E), highlighting its potential as a therapeutic target for HCC treatment.

### 3.4 Immune infiltration patterns in HCC patients with different risk profiles

**FIGURE 4:**
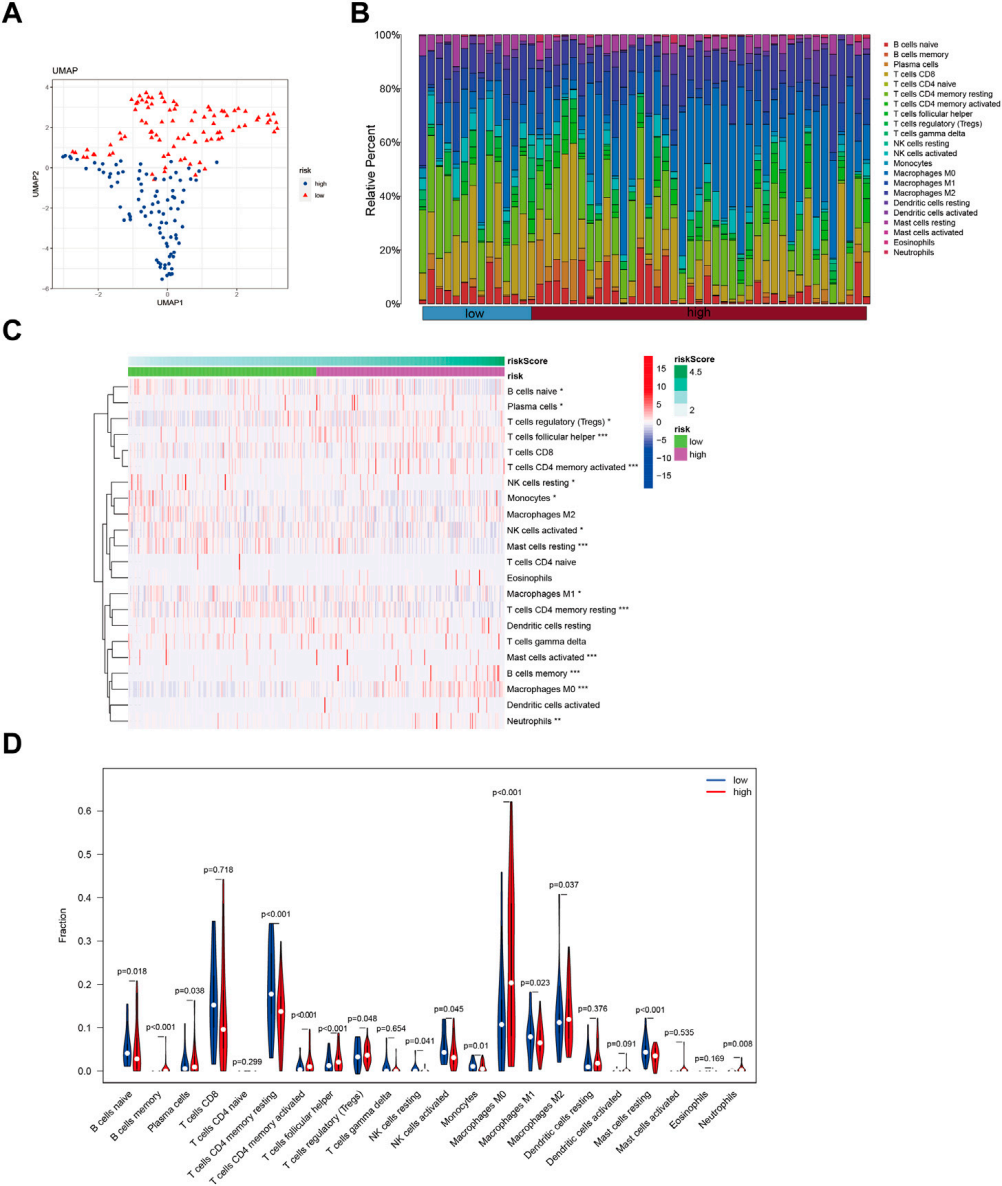
Identify immune landscape of HCC based on glycolysis-associated signature. **(A)** UMAP demonstrates different immune profiles among HCC subgroups. **(B)** Proportion of immune cells in HCC tissues. **(C and D)** Differences in immune infiltration between HCC subgroups.

In this study, we utilized a set of 10 GMGs to explore immune infiltration patterns in heterogeneous risk profiles of patients with hepatocellular carcinoma. The Lasso method was applied to conduct dimensionality reduction and clustering analysis, and our results, depicted in Figure 4A, successfully distinguished HCC patients across different risk categories. To further evaluate the immune cell landscape, we assessed immune cell abundance across different risk scores (Figure 4B). Our findings revealed a higher infiltration of regulatory T Cells (Tregs) and macrophage M0 in high-risk patients, compared to that of macrophage M1 (Figures 4C,D).

**FIGURE 5:**
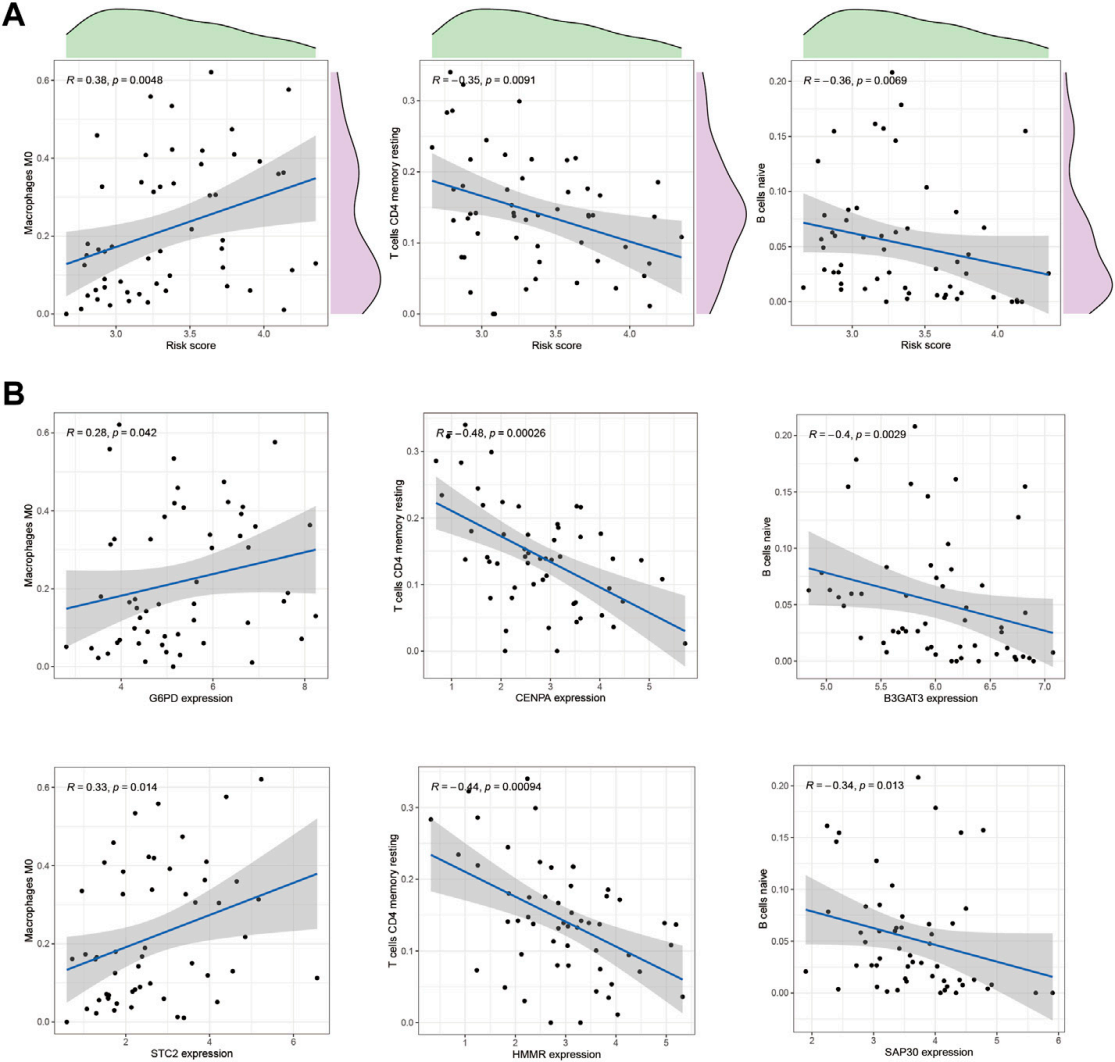
Correlation with immune cells. **(A)** The correlation between the risk score and immune cell types, such as Macrophage M0 cells and CD4 memory resting T Cells, in HCC tissues was analyzed. **(B)** In addition, the relationship between immune cells and GMGs was also examined.

**FIGURE 6:**
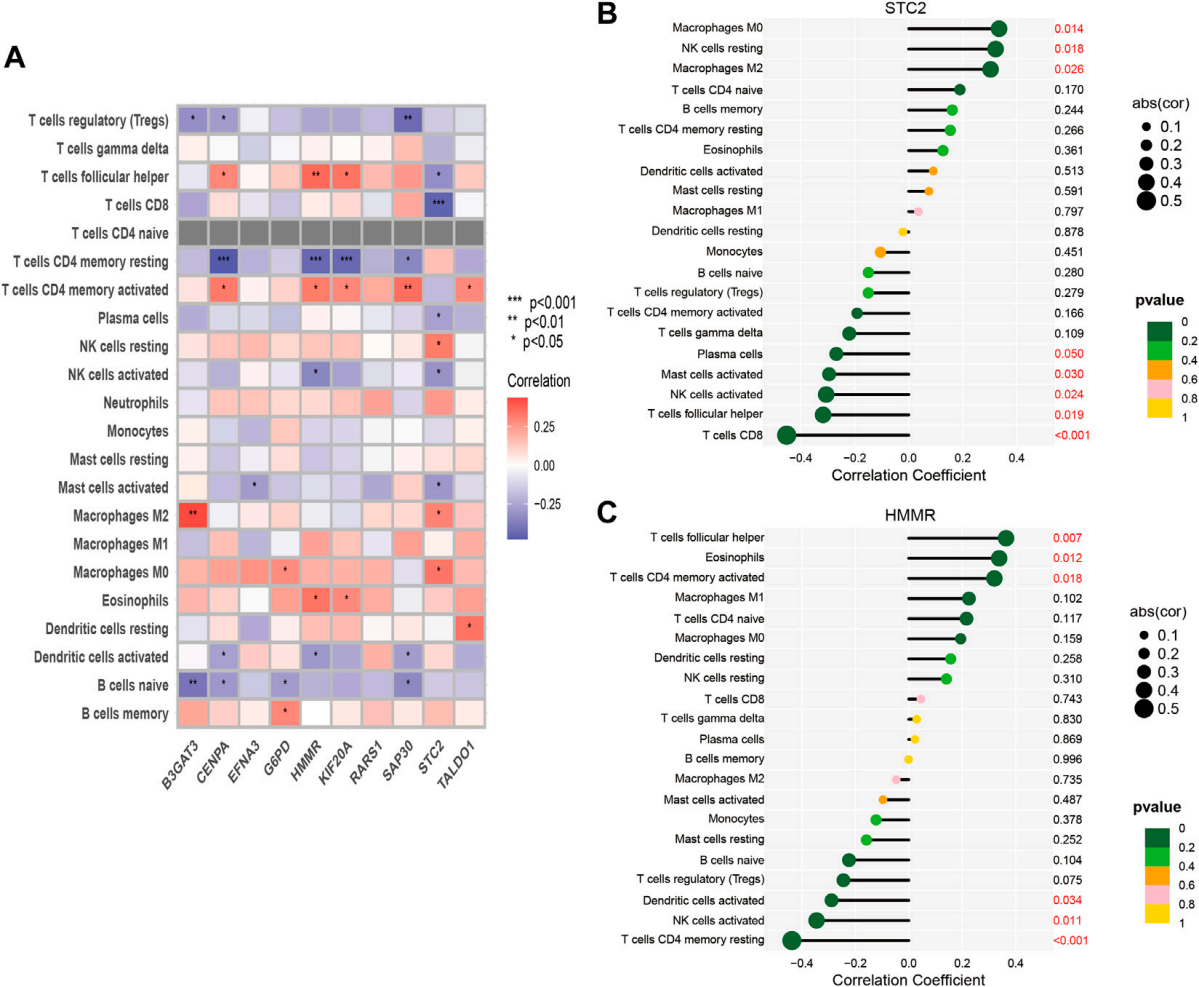
Correlation between immune cells and 10-GMGs. **(A)** Heatmap was used to show the correlation between immune cells and 10-glycolysis metabolic genes (10-GMGs). **(B and C)** Bar plots were used to illustrate the relationship between STC2 and HMMR with immune cell infiltration.

One of the GMGs investigated in this study was G6PD, a crucial metabolic enzyme involved in glycolysis. Our results, presented in Figure 5, indicated a positive correlation between G6PD and STC2 expression levels and the infiltration of M2 macrophages. Conversely, the expression levels of CENPA and HMMR were negatively correlated with the presence of CD4 memory resting T Cells, while B3GAT3 and SAP30 expression levels were negatively correlated with naive B Cells. Our results were consistent with the riskscore distribution, as shown in Figure 5A. Additionally, significant associations were found between STC2 and HMMR with multiple immune cell types, as revealed by our correlation analysis between the ten GMGs and various immune cell types (Figures 6A,B).

### 3.5 Relationship between GMG expression and immunotherapy response

**FIGURE 7:**
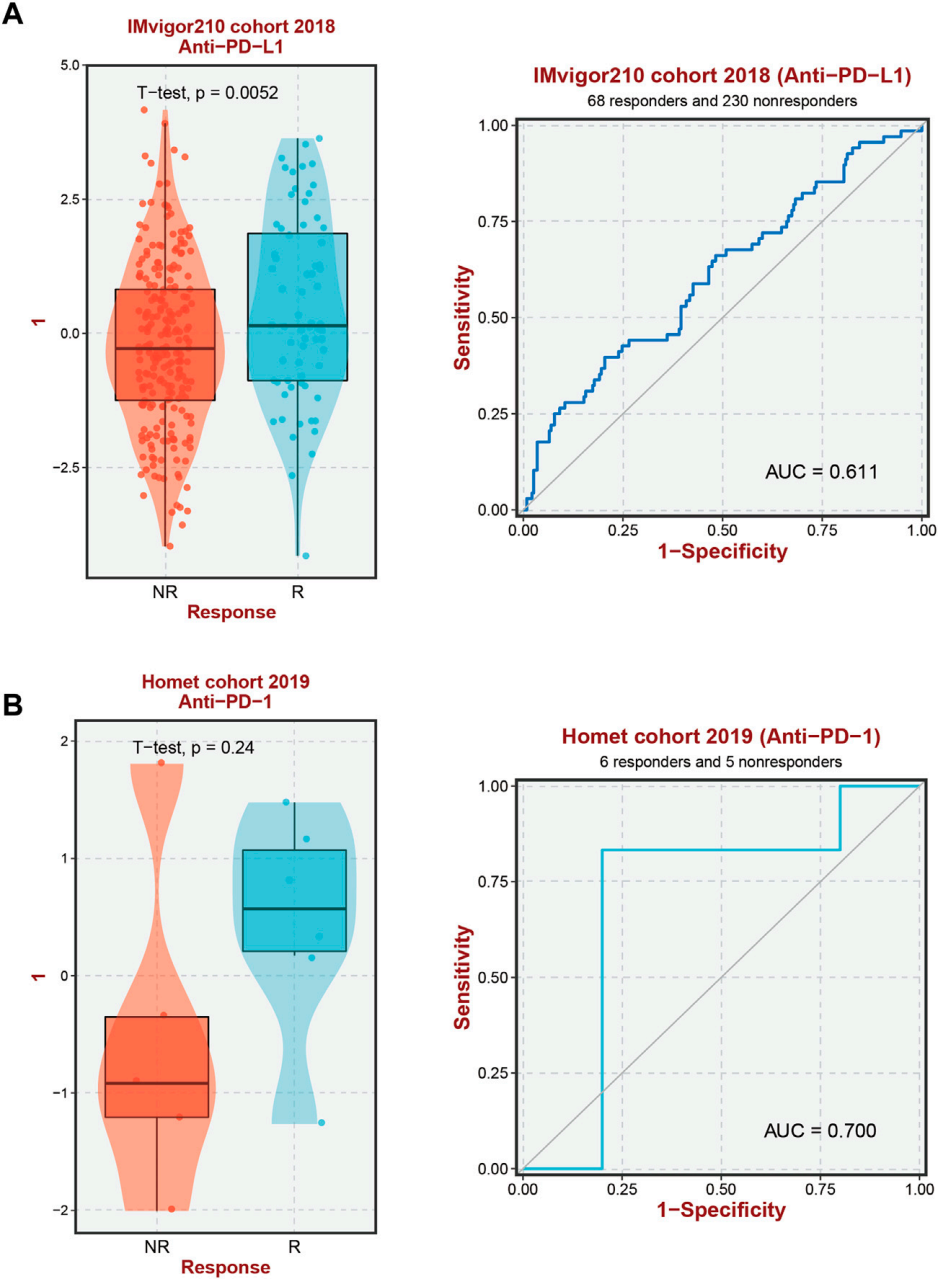
Immunotherapy response prediction. **(A)** Prediction of immune therapy response to anti-PD-L1 treatment in HCC patients based on 10-GMGs. **(B)** Prediction of immune therapy response to anti-PD-1 treatment in HCC patients based on 10-GMGs.

In our previous investigation, we identified unique immunological microenvironments in patients with high-risk and low-risk HCC. Specifically, the high-risk group exhibited elevated levels of Macrophage M0 and Tregs, which have immunosuppressive properties. Effective activation of CD4 memory T Cells is essential for a positive response to immune therapy, as these cells display varying degrees of sensitivity to immunotherapeutic modalities. Notably, we observed a substantial increase in the expression of ten GMGs in HCC patients who exhibited positive responses to Anti-PD-L1 and Anti-PD-1 immunotherapies. This discovery suggests that these genes may serve as potential biomarkers for predicting the effectiveness of immune checkpoint blockade (ICB) treatment in HCC patients (Figures 7A,B).

**FIGURE 8:**
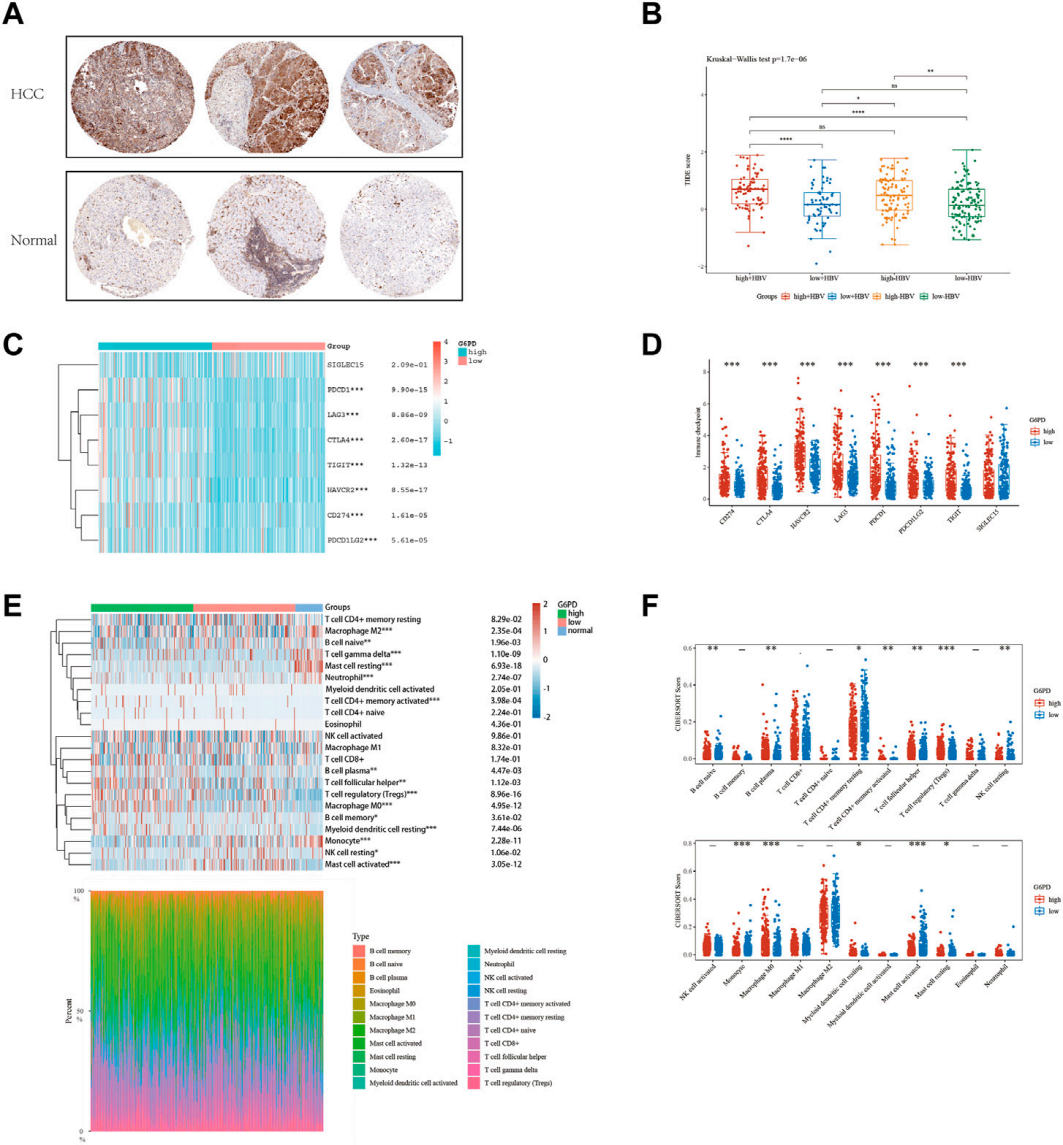
The level of immune checkpoint in HCC subtypes. **(A)** The HPA online database shows that G6PD is overexpressed in HCC tissues. **(B)** HBV infection does not affect the effectiveness of immunotherapy. **(C and D)** There are differences in the expression of immune checkpoint markers between the high-risk and low-risk groups of HCC. **(E and F)** CIBERSORT analysis revealed differences in immune infiltration between the subgroups.

The glycolytic pathway, with G6PD as a key enzyme, plays a critical role in the metabolism of cancerous cells. We detected a marked increase in G6PD protein expression in the tissues of patients with HCC (Figure 8A). To assess the effectiveness of immune checkpoint blockade (ICB) in HCC patients at high and low risk, we utilized the TIDE algorithm, an innovative approach that integrates G6PD expression levels and HBV infection factors (Figure 8B). Our analysis revealed a positive correlation between elevated G6PD expression levels and immune response score, independent of HBV status. We further classified HCC patients into high and low G6PD expression groups and observed a significant upregulation of immune checkpoint markers, including PDCD1 and CD274, in the high G6PD expression group (Figures 8C,D). Additionally, our Cibersort analysis revealed substantial differences in immune cell infiltration levels among normal, low-risk, and high-risk HCC patient tissue samples (Figures 8E,F). Taken together, our results suggest that G6PD expression levels may serve as a promising biomarker for predicting response to ICB in HCC patients.

### 3.6 Stemness scores in hepatocellular carcinoma patients

**FIGURE 9:**
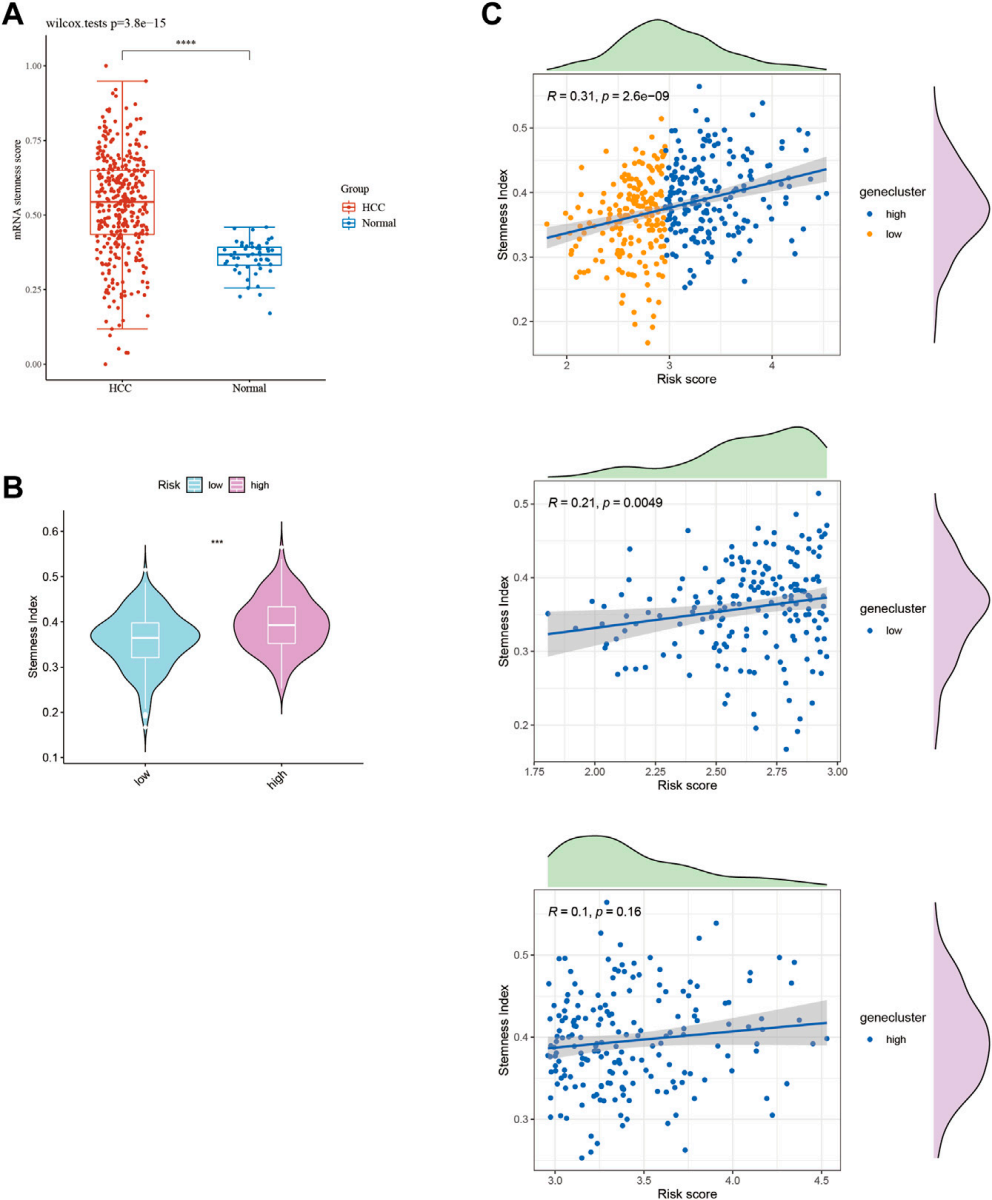
Stemness score in HCC subtypes. **(A)** Stemness score between HCC tissues and normal tissues. **(B)** Stemness score between HCC subtypes. **(C)** Stemness index of HCC patients scored by 10-GMGs.

HCC exhibits significant heterogeneity, which affects both tumor progression and treatment response. To investigate the stemness phenotype as a contributing factor to this heterogeneity, we assessed stemness scores in HCC patients stratified by risk level. Our analysis revealed significantly higher stemness ratings in HCC patients than in healthy liver tissue (Figure 9A). Moreover, high-risk HCC patients displayed significantly elevated stemness scores compared to low-risk individuals (Figure 9B). Notably, even among low-risk patients, we observed a significant positive correlation between risk ratings and stemness index (R = 0.31; Figure 9C). Our results underscore the pivotal role of stemness in HCC pathogenesis and suggest that targeting stemness may represent a promising therapeutic approach for HCC. Nevertheless, a better understanding of the underlying mechanisms linking stemness to HCC risk stratification is needed and warrants further investigation.

### 3.7 Anticipation of drug responsiveness and authentication

**FIGURE 10:**
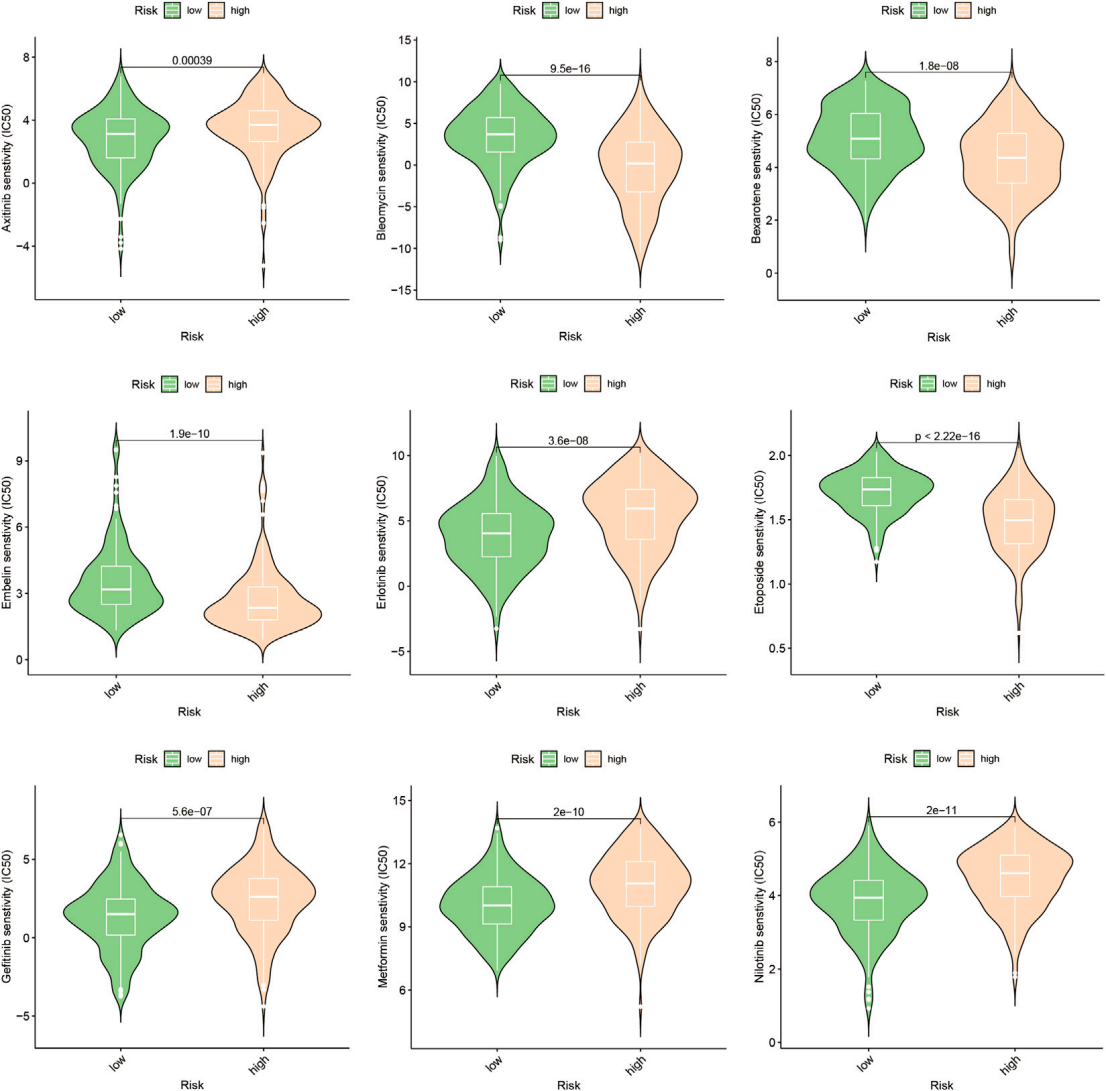
Drug sensitivity prediction. Drug sensitivity in patients with different risks of HCC.

**FIGURE 11:**
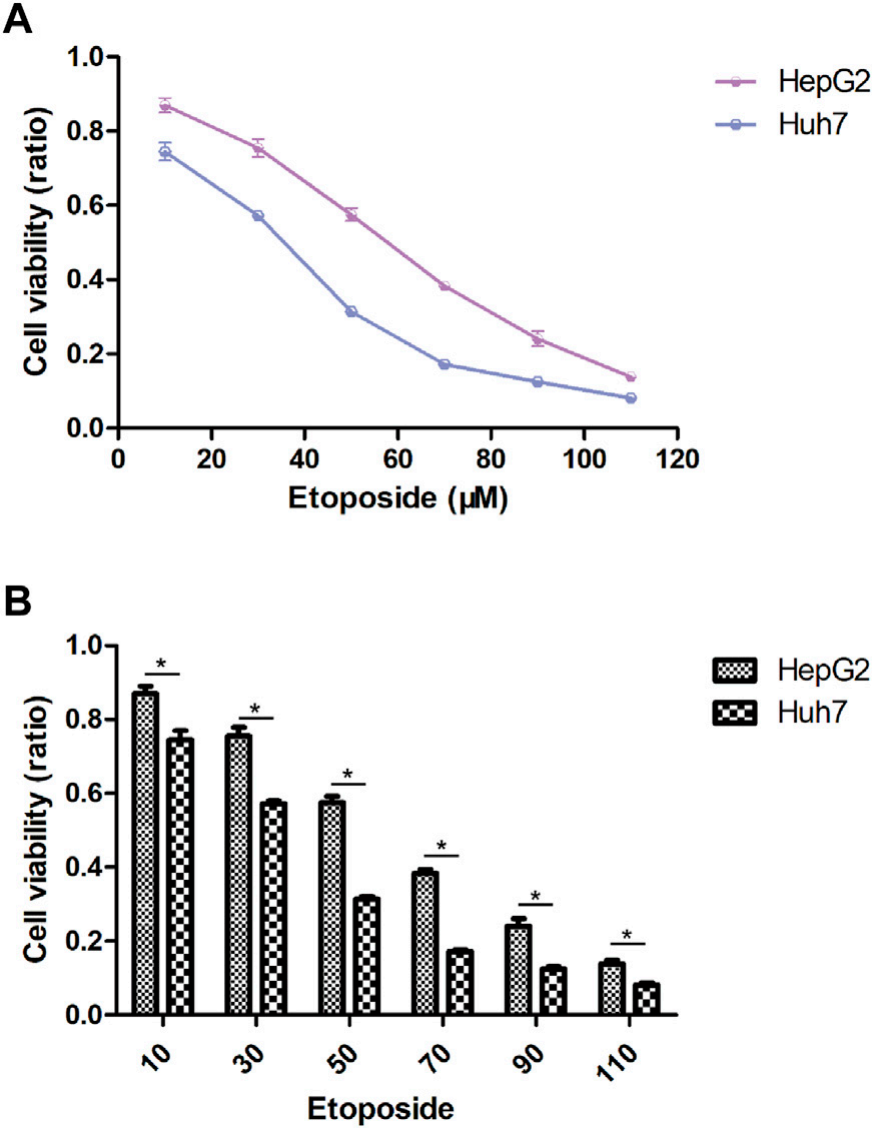
Drug sensitivity in HCC cell lines. **(A)** IC50 after exposure to etoposide. **(B)** Etoposide sensitivity of HCC cell lines in different risk scores.

In this study, we aimed to evaluate the efficacy of personalized therapy for managing HCC in patients with diverse risk profiles by assessing variations in chemotherapeutic drug sensitivity. Specifically, we measured the IC50 concentrations of nine chemotherapeutic agents in HCC subgroups categorized according to high and low risk scores, as illustrated in Figure 10. Our analysis revealed significant inter-subgroup heterogeneity in IC50 values, with Etoposide exhibiting the most pronounced disparity. Additionally, we confirmed the enhanced susceptibility of HCC patients with high-risk scores to Etoposide, as demonstrated in Supplementary Table S1. We calculated the corresponding risk scores for hepatocellular carcinoma (HCC) cell lines based on the coefficients derived from the prognostic model of 10-gene expression signatures (10-GMGs). According to the computational results, the risk score for Huh7 was significantly higher than that for HepG2, indicating a greater risk associated with Huh7. To assess drug treatment efficacy, we selected Huh7 and HepG2 cells as representatives of the subgroups with high and low risk scores, respectively, and determined their IC50 values for Etoposide using the CCK-8 assay (Figure 11A). Notably, the IC50 of Etoposide in Huh7 cells was significantly lower than that in HepG2 cells, lending support to the potential therapeutic benefits of Etoposide chemotherapy for patients with high-risk scores, as identified by our analysis. These findings are consistent with our drug sensitivity prediction results (Figure 11B) and underscore the promise of personalized therapy in improving the efficacy of HCC treatment across varying risk scores.

## 4 Discussion

Accurate and timely diagnosis of HCC is essential for optimizing patient outcomes. However, the high heterogeneity of HCC tissue poses a significant challenge to the accuracy of current clinical classification systems (Sung et al., 2021). Moreover, the complex molecular mechanisms involved in HCC create obstacles for identifying effective therapeutic targets (Llovet et al., 2018; Cheng et al., 2020; Soltani et al., 2021). The Warburg effect, a phenomenon in which tumor cells rely on aerobic glycolysis to evade apoptosis, plays a crucial role in maintaining cellular function, particularly in malignancy (Chen et al., 2015; Porporato et al., 2018; Guo et al., 2022). As the liver plays a vital role in energy metabolism, HCC tumorigenesis is inextricably linked to glycolysis. Previous studies have demonstrated that HCC growth, metastasis, and resistance to treatment are closely associated with glycolytic metabolism (Park et al., 2021; Wang et al., 2022b; Zhou et al., 2022; Dou et al., 2023; Vashishta et al., 2023). Therefore, the construction of accurate prognostic models utilizing machine learning techniques that incorporate glycolysis-related genes is essential for precise diagnosis, individualized therapy, and the prediction of clinical outcomes in HCC patients (Toh et al., 2023).

To identify potential prognostic markers for HCC, our study employed a screening strategy utilizing a panel of 200 metabolic genes linked to glycolysis. By conducting a differential gene expression analysis, we identified 10 GMGs that were significantly correlated with HCC prognosis. Subsequently, we employed this subset to establish a predictive model for HCC prognosis (Figure 1C). Our model effectively predicted overall mortality rates among HCC patients over different time periods, including 1, 3, 5, and 7 years following diagnosis. These results suggest that our GMGs-based prediction model may have significant clinical applications in the decision-making process for HCC patients. Moreover, our findings indicate that GMGs may serve as a promising new class of prognostic biomarkers for HCC.

The tumor microenvironment (TME) plays a crucial role in the pathogenesis of cancers (Song et al., 2021; Li et al., 2023), and recent studies have demonstrated the promotion of malignancy within TME via exosome-mediated signaling (Wu et al., 2019; Gong et al., 2022). Reliable prognostic indicators in HCC include patterns of immune infiltration within the TME (Kamil and Rowe, 2018; Feng et al., 2022), with regulatory T Cells (Tregs) contributing to an immunosuppressive milieu that supports cancer cell survival while impeding immune surveillance (Wang et al., 2021). In addition, increasing evidence indicates that neutrophils serve as key immunosuppressive regulators in the TME of various malignancies, including HCC (Geh et al., 2022). Our study reveals a significant association between elevated levels of neutrophil and Treg infiltration and increased GMG expression, as demonstrated in Figure 4C. Although M2 macrophages have been extensively studied for their role in promoting tumor development in HCC, recent investigations have highlighted the ability of M0 macrophages to inhibit T cell-mediated anti-tumor responses (Vinnakota et al., 2017). For instance, the miR-149-5p/MMP9 signaling pathway has been identified as a mechanism through which M2 macrophages facilitate HCC cell motility and invasion (Liu et al., 2020).

The metabolic shift towards glycolytic metabolism that leads to lactate accumulation and polarization of macrophages towards an M2-like phenotype is a defining characteristic of cancer development (Zhang and Li, 2020; Jiang et al., 2022). This change may explain the differential enrichment of M0 and M2 macrophages observed in individuals with higher GMGs expression, as depicted in Figure 4D. Cancer therapy often involves harnessing the immune system to detect and eradicate cancer cells. Numerous immunotherapy approaches have been explored, including checkpoint inhibitors, adoptive cell transfer, and cancer vaccines (Sangro et al., 2021; Jin et al., 2022; Llovet et al., 2022; Zhao et al., 2022). M0 macrophages in the tumor microenvironment have been shown to inhibit T cell-mediated anti-tumor responses and to secrete tumor-promoting factors (Vinnakota et al., 2017). PD-1 and PD-L1 have close associations with macrophages (Liu et al., 2018). Additionally, lactic acid can elevate PD-1 expression in Tregs within glycolytic tumor microenvironments (Kumagai et al., 2022). Our study revealed higher PD-1 and PD-L1 mRNA expression levels in the high GMGs group than in the low GMGs group (Figures 8C,D), which could indicate the presence of Tregs and macrophages. This finding may account for the improved response to anti-PD-1 and anti-PD-L1 therapy observed in the high GMG group of HCC patients (Figure 7).

Enhanced glycolysis plays a crucial role in the growth and progression of liver cancer. Through glycolysis, liver cancer cells can rapidly generate a large amount of energy and biosynthetic precursors to meet their aberrant proliferative demands. Stemness refers to the capacity of tumor cells for self-renewal and unrestricted differentiation. The enhancement of glycolysis is associated with the presence and proliferation of stem-like liver cancer cells. Stem-like liver cancer cells tend to sustain their stemness state by producing lactate through glycolysis. This metabolic characteristic enables stem-like liver cancer cells to resist conventional therapies such as radiation and chemotherapy. Our study revealed that high-risk HCC patients exhibit higher stemness (Figure 9B). Additionally, as the risk score increases, the stemness of low-risk HCC patients also increases correspondingly, while the stemness of high-risk patients remains relatively unchanged (Figure 9C).

In order to gain a comprehensive understanding of the etiology of HCC, it is crucial to investigate not only the interactions between tumor cells and immune cells but also the dysregulation of signaling pathways within tumor cells, as these factors can profoundly impact the initiation and progression of HCC (Llovet et al., 2018). This study identified several pathways, including cytoplasmic translation, fibrinolysis, blood microparticles, and cytosolic ribosomes, that were significantly enriched in HCC patients with elevated gene module groups (GMGs) and may therefore affect their response to chemotherapy (Figures 3C,E). Based on the identification of 10 GMGs, we also identified nine potential therapeutic agents that could be effective for certain subtypes of HCC (Figure 10). To validate our predictions, we used etoposide as a test substance and a classification system based on the transcript levels of GMGs in HCC cell lines, differentiating between Huh7 cells with high GMG levels and HepG2 cells with low GMG levels. Our results revealed that Huh7 cells exhibited a lower IC50 after exposure to various doses of etoposide (Figure 11A). Moreover, Huh7 cells exposed to etoposide under the same therapeutic conditions and dosage exhibited greater cytotoxicity than HepG2 cells (Figure 11B). These findings not only demonstrate the effectiveness of our methodology but also lend strong support to the drug sensitivity predictions we made using GMGs.

In recent years, there has been a growing emphasis on investigating the potential association between glycolytic metabolism-related genes and tumor development. Emerging evidence has highlighted the pivotal role of glycolytic gene expression levels in shaping the tumor microenvironment, which in turn modulates the efficacy of chemotherapy and immunotherapy for HCC patients. Therefore, personalized treatment regimens tailored to the individual glycolytic profiles of patients are of paramount importance. Gene expression profiling has been demonstrated in numerous studies to be a valuable tool for accurate classification of tumor tissue (Jin et al., 2021; Liu et al., 2022; Zhong et al., 2022).

HCC is a malignant tumor characterized by pronounced intratumoral heterogeneity, which is closely associated with tumor growth and therapeutic resistance, and has been linked to an increased risk of treatment failure and unfavorable prognosis. Cancer stem cells (CSCs) are a subset of cells within tumors that possess unique self-renewal and multipotency capabilities, and have been implicated in driving tumor heterogeneity, as well as contributing to treatment resistance and disease recurrence. Thus, the role of stemness in HCC heterogeneity was investigated by evaluating stemness levels in patients with varying risk scores. As illustrated in Figure 9A, the stemness score of HCC patients was significantly higher than that of normal liver tissue. Furthermore, high-risk HCC patients exhibited a markedly elevated stemness score compared to their low-risk counterparts, as indicated by the results presented in Figure 9B. Notably, our analysis revealed a positive correlation between risk score and stemness index in HCC patients, as shown in Figure 9C. These data highlight the potential of our 10-gene model as an accurate predictor of stemness index in HCC patients, and suggest its potential use in identifying new therapeutic targets for intervention in high-risk populations.

Our study aimed to examine the heterogeneity and stemness of HCC patients and the corresponding changes in their microenvironment through patient stratification based on gene expression levels within the glycolysis pathway. Our findings demonstrated considerable differences in immune infiltration patterns and prognosis among HCC patients with distinct levels of glycolytic metabolic gene expression. Remarkably, we discovered a strong association between the degree of dryness and the probability of survival in HCC patients. We developed a glycolysis-related model using ten genes, which displayed a high degree of accuracy in predicting patient outcomes. This model has significant implications as a prognostic tool for HCC. To reinforce the clinical relevance of our model, we conducted cell toxicity experiments to assess its capacity to predict chemotherapeutic sensitivity. Our results provide crucial information for physicians to make informed decisions regarding HCC treatment.

## 5 Conclusion

Tumor heterogeneity in HCC is primarily attributed to stemness, which is, in turn, critically regulated by glycolysis. However, the clinical significance of glycolysis-related metabolic genes in HCC prognosis is still poorly understood. Thus, the objective of this investigation is to identify crucial GMGs and develop a robust prognostic model for HCC. By employing receiver operating characteristic (ROC) curves, we developed a 10-GMG-based risk model that exhibits high predictive accuracy, which was subsequently validated. Furthermore, two distinct GMG-related subtypes were identified, exhibiting significant differences in tumor stemness, immune landscape, and prognostic stratification, indicating a considerable degree of heterogeneity in HCC risk. Notably, these findings also suggest that patterns in immunotherapy and chemotherapy responses may be associated with HCC heterogeneity and stemness diversity among patients. *In vitro* validation confirmed the predictive value of our model for drug response. In summary, this study provides clinicians with a potential strategy for precision medicine targeting HCC heterogeneity by utilizing the 10-GMGs model.

## Data Availability

The datasets presented in this study can be found in online repositories. The names of the repository/repositories and accession number(s) can be found in the article/Supplementary Material.
