# Supplementary figures and images for: Multi-omic analysis of glycolytic signatures: exploring the predictive significance of heterogeneity and stemness in immunotherapy response and outcomes in hepatocellular carcinoma
Source: Front Mol Biosci. 2023 Jun 7;10:1210111. doi: 10.3389/fmolb.2023.1210111 (PMC10282758; doi:10.3389/fmolb.2023.1210111)

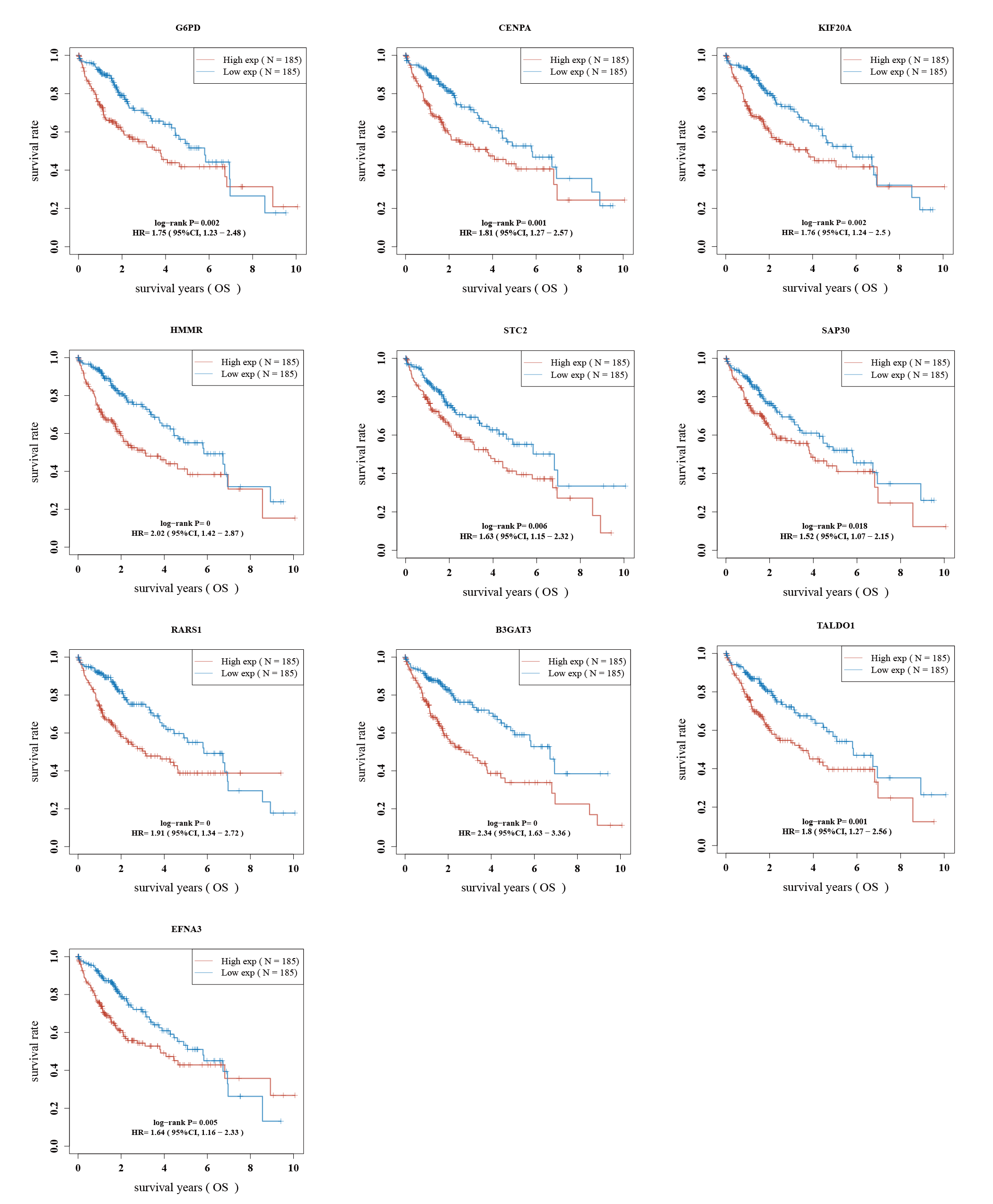

Supplement: Supplementary file 1 [file Image1.TIF]
